# Aflatoxin B1 degradation during co-cultivation of *Aspergillus flavus* and *Pleurotus ostreatus* strains on rice straw

**DOI:** 10.1007/s13205-014-0228-7

**Published:** 2014-06-11

**Authors:** Arijit Das, Sourav Bhattacharya, Muthusamy Palaniswamy, Jayaraman Angayarkanni

**Affiliations:** 1 Department of Microbiology, Karpagam University, Coimbatore, 641021 Tamil Nadu India; 2 Department of Microbiology, Centre for Advanced Studies in Biosciences, Jain University, Bangalore, 560019 Karnataka India; 3 Department of Microbial Biotechnology, Bharathiar University, Coimbatore, 641046 Tamil Nadu India

**Keywords:** Aflatoxin B1, *Pleurotus ostreatus*, Co-cultivation, Rice straw

## Abstract

Aflatoxin B1 (AFB1) produced by *Aspergillus flavus* is known to have carcinogenic and teratogenic effects on animal health. Accidental feeding of AFB1-contaminated rice straw may be detrimental to dairy cattle. White-rot basidiomycetous fungus *Pleurotus ostreatus* can grow on different agronomic wastes by synthesizing different ligninolytic enzymes. These extracellular enzymes are capable of degrading many environmentally hazardous compounds including AFB1. The present study examines the ability of different strains of *P. ostreatus* to degrade AFB1 in contaminated rice straw. Different strains of *A. flavus* were inoculated on rice straw for AFB1 production. The moldy straw was then subjected to co-cultivation by different strains of *P. ostreatus*. The extent of AFB1 degradation was determined by high performance liquid chromatography. Results indicated the presence of AFB1 in the moldy straw samples at levels of 27.95 ± 0.23 and 21.26 ± 0.55 µg/g of dry substrate for *A. flavus* MTCC 2798 and *A. flavus* GHBF09, respectively. Co-cultivation of *P. ostreatus* strains on AFB1-contaminated rice straw revealed their ability to rapidly colonize the substrate by profuse hyphal ramification. Highest degradation of AFB1 (89.41 %) was recorded in the straw containing co-cultures of *A. flavus* MTCC 2798 and *P. ostreatus* GHBBF10. Natural isolate *P. ostreatus* GHBBF10 demonstrated higher AFB1-degradation potential than *P.ostreatus* MTCC 142. This basidiomycete strain can be further exploited to effectively degrade moderate concentrations of AFB1 in contaminated moldy rice straw.

## Introduction

Mycotoxins are highly toxic fungal metabolites which have adverse effects on human and animal health. Among the mycotoxins, aflatoxin B1 (AFB1) is extremely hazardous for being mutagenic, carcinogenic and teratogenic (Khoshpey et al. 2011). AFB1 is produced by strains of *Aspergillus flavus* and *A. parasiticus* on a variety of agricultural commodities. Animal feeds and forages may become contaminated with AFB1 in the fields and/or during storage under conditions conducive for fungal growth (Streit et al. 2012). In the tropical countries like India, rice (paddy) straw is often used as cattle feed. However, consumption of AFB1-contaminated straw can lead to aflatoxicosis in cattle and eventually lead to transmission of these toxins or their metabolites into the milk of dairy animals (Bennett and Klich 2003).

White-rot basidiomycetous fungus such as *Pleurotus ostreatus* is able to transform AFB1 into less toxic metabolites (Guan et al. 2008). Under natural condition, *P. ostreatus* grows on dead wood but can be easily cultivated on different agro-industrial wastes (Sánchez 2010). It produces different oxidative [laccase and manganese peroxidase (MnP)] and hydrolytic (cellulase, xylanase and tannase) enzymes which are involved in utilization of lignocellulosic substrates (da Luz et al. 2012).

Degradation of AFB1 by white-rot fungi through the production of extracellular lignin modifying enzymes was previously investigated (Cohen et al. 2002). Due to lack of substrate specificity, laccase can degrade many xenobiotic compounds. A correlation between laccase activity and AFB1 degradation was previously reported by Alberts et al. (2009). Though some studies on AFB1 degradation have been conducted, very few scientific literatures are available on AFB1 degradation by *P. ostreatus* in *A. flavus*-contaminated moldy rice straw. Among *P. ostreatus*, different strains may vary in their ability to degrade AFB1. Selection of a potent basidiomycete strain may facilitate effective degradation of this hazardous toxin. Therefore, the present study was conducted to assess the ability of different strains of *P. ostreatus* to degrade AFB1 in contaminated rice straw.

## Materials and methods

### Chemicals and reagents

AFB1 standard was procured from Sigma (USA). All other analytical grade laboratory chemicals were purchased from S D Fine-Chem Ltd. (Mumbai, India) and Nice Chemicals (Kochi, India).

### Source of fungal strains

*A.flavus* MTCC 2798 and *P.ostreatus* MTCC 142 were obtained from Microbial Type Culture Collection and Gene Bank, Chandigarh, India. An aflatoxigenic fungal strain identified as *A. flavus* GHBF09 (GenBank accession number KC987360) was isolated from rhizosphere soil (Das et al. 2013a). A basidiomycetous fungal strain identified as *P.ostreatus* GHBBF10 (GenBank accession number KC987361) was isolated from a decomposing tree trunk (Das et al. 2013b). Cultures of *A.flavus* and *P.ostreatus* were maintained on potato dextrose agar and glucose yeast extract agar plates, respectively.

### Substrate preparation

Rice (paddy) straw was obtained from the local market in Bangalore. The straw was washed several times under running tap water, dried in hot air oven at 80 °C and cut into approximately 1 cm pieces. This was used as the substrate for co-cultivation. Dry straw (1 g) was filled in a glass culture tube measuring 2.5 cm in diameter and 15 cm long, moistened with 5 ml supplemented mineral salt solution containing (g/l) lactose, 10; yeast extract, 5; KH_2_PO_4_, 0.2; MgSO_4_·7H_2_O, 0.1; NH_4_Cl, 0.3; CaCO_3_, 1.0 and distilled water, at initial pH 5.8 and autoclaved (Das et al. 2013b).

### Co-cultivation of *A. flavus* and *P. ostreatus* strains

Degradation of AFB1 during co-cultivation of *A. flavus* and *P. ostreatus* strains on rice straw was investigated. Duplicate sets of four culture tubes containing the substrate were labeled. Two tubes were inoculated with spore suspension of *A. flavus* MTCC 2798 and two other tubes with *A. flavus* GHBF09. The inoculated sets of tubes were incubated at 28 ± 2 °C for 15 days for AFB1 production. Post incubation, one set of tubes was subjected to AFB1 analysis using high performance liquid chromatography (HPLC). In the second set, two alternate tubes containing moldy straw with live *A. flavus* cultures were aseptically inoculated with two mycelial plugs of *P. ostreatus* MTCC 142, while the other two tubes were inoculated with *P.ostreatus* GHBBF10. This set of inoculated tubes was incubated at 30 ± 2 °C for 15 days in dark. Mycelial growth in these tubes was carefully monitored daily. After 15 days of incubation, the level of residual AFB1 present in the tubes was determined by HPLC. A sterile uninoculated tube containing the substrate was maintained separately throughout the study for sterility check.

### Extraction of AFB1

The extraction of AFB1 was performed using AOAC protocols (AOAC International 2000) with few modifications. Following incubation, the substrate colonized with fungal mycelia was homogenized in a blender with 20 ml of 90 % (v/v) methanol and filtered through coarse filter paper. To the filtrate equal volume of methylene chloride was added and AFB1 was partitioned by shaking vigorously for 15 min. The extraction procedure was repeated with the aqueous fraction. The methylene chloride fractions were pooled and concentrated in hot air oven at 40 °C overnight.

### Analysis of AFB1 using HPLC

The concentrated residue was reconstituted in 1 ml of acetonitrile and filtered through 0.25 μ nylon membrane filter (Axiva Sichem Biotech, Delhi, India). The working standard solution of AFB1 (concentration of 10 μg/ml) was prepared using acetonitrile. Working standard (20 μl) containing 0.2 μg of standard AFB1 was injected into the HPLC system (Waters, USA, model number-2487, with Dual λ absorbance UV detector and binary pump system, model number-1525). A reverse phase, YMCA Triart C18 column (150 × 4.6 mm, 3 μm) was used. The mobile phase consisted of acetonitrile-methanol-water (1:1:2, v/v/v). A total run time of 14 min was maintained at a flow rate of 0.8 ml/min under ambient temperature. The absorbance of AFB1 was determined at 360 nm. Area under AFB1 absorbance peak was used to estimate its concentration and also the percentage of degradation using the following formula (Guan et al. 2010):

$$\text{Percentage of AFB1 degradation}=[\left(C_{\text{i}}-C_{\text{f}}\right)/C_{\text{i}}]\times 100$$

where, *C*_i_ is the initial concentration of AFB1 and *C*_f_ is the final concentration of AFB1.

### Statistical analysis

The experiment was conducted in triplicate. The data were analyzed using single factor analysis of variance (ANOVA) and graphically presented as mean ± SD (*n* = 3). ANOVA was performed using Microsoft Excel 2007. *P* values <0.05 were considered significant with a confidence limit of 95 %.

## Results and discussion

In the present study, co-cultivation of *A. flavus* and *P. ostreatus* strains on rice straw was carried out to understand the practical applicability of the basidiomycetous fungus in AFB1 degradation in rice straw which may be contaminated with AFB1 in agricultural fields.

### Growth of *A. flavus* strains on rice straw and AFB1 production

**Fig. 1:**
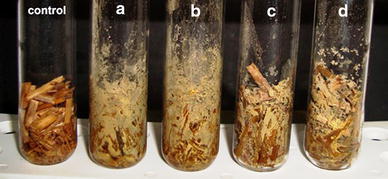
Mycelial growth of *A. flavus* in tubes containing moist rice straw as substrate.*a*,*b* Tubes inoculated with *A. flavus* MTCC 2798.*c*,*d* Tubes inoculated with *A. flavus* GHBF09. A sterile uninoculated tube (control) is shown on the *left*

After 15 days of incubation, rice straw inoculated with the tested strains of *A. flavus* turned completely moldy due to profuse mycelial growth and sporulation of the fungi (Fig. 1). HPLC analysis of these moldy straw samples revealed the presence of AFB1 at levels of 27.95 ± 0.23 and 21.26 ± 0.55 µg/g of dry substrate for *A. flavus* MTCC 2798 and *A. flavus* GHBF09, respectively. Comparatively, *A. flavus* MTCC 2798 demonstrated higher production of AFB1. Zain (2011) suggested that among various factors, the ability to produce AFB1 in foods and feeds is also dependent on fungal strain specificity and strain variation. Moreover, the presence of elevated moisture content and high C/N ratio in rice straw would have facilitated increased AFB1 production from the tested strains of *A. flavus* (Abdulla 2007).

### Growth of *P. ostreatus* strains on moldy rice straw

The growth of oyster mushroom generally depends on the type of substrate used for its cultivation. Generally, rice straw supports higher yields of oyster mushrooms compared to wheat straw under identical cultivation conditions (Zhang et al. 2002). The presence of high initial moisture content (83 %) within the straw fibers together with utilizable lignin and cellulose would have stimulated abundant mycelial growth of *P. ostreatus* through the synthesis of lignocellulolytic enzymes like laccase and cellulase. An earlier study reported larger fruiting bodies in *P. eryngii* when cultivated on rice straw (Moonmoon et al. 2010).

**Fig. 2:**
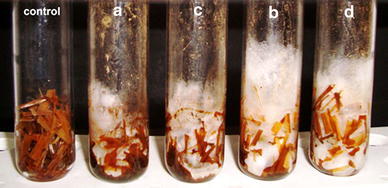
Tubes containing moist rice straw with co-cultures of *A. flavus* and *P. ostreatus* strains.*a* Tube inoculated with *A. flavus* MTCC 2798 + *P. ostreatus* MTCC 142.*b* Tube inoculated with *A. flavus* MTCC 2798 + *P. ostreatus* GHBBF10.*c* Tube inoculated with *A. flavus* GHBF09 + *P. ostreatus* MTCC 142.*d* Tube inoculated with *A. flavus* GHBF09 + *P. ostreatus* GHBBF10. A sterile uninoculated tube (control) is shown on the *left*

Co-cultivation of *P. ostreatus* strains on rice straw already containing *A. flavus* culture revealed the ability of *P. ostreatus* strains to rapidly colonize the substrate. Following incubation, the tubes inoculated with *P. ostreatus* MTCC 142 showed moderate hyphal proliferation, whereas, the tubes inoculated with *P. ostreatus* GHBBF10 demonstrated profuse mycelial growth (Fig. 2). This basidiomycete strain might have synthesized higher titres of extracellular enzymes, which would have resulted in better utilization of the lignocellulosic substrate.

### AFB1 degradation during co-cultivation of fungal strains

**Fig. 3:**
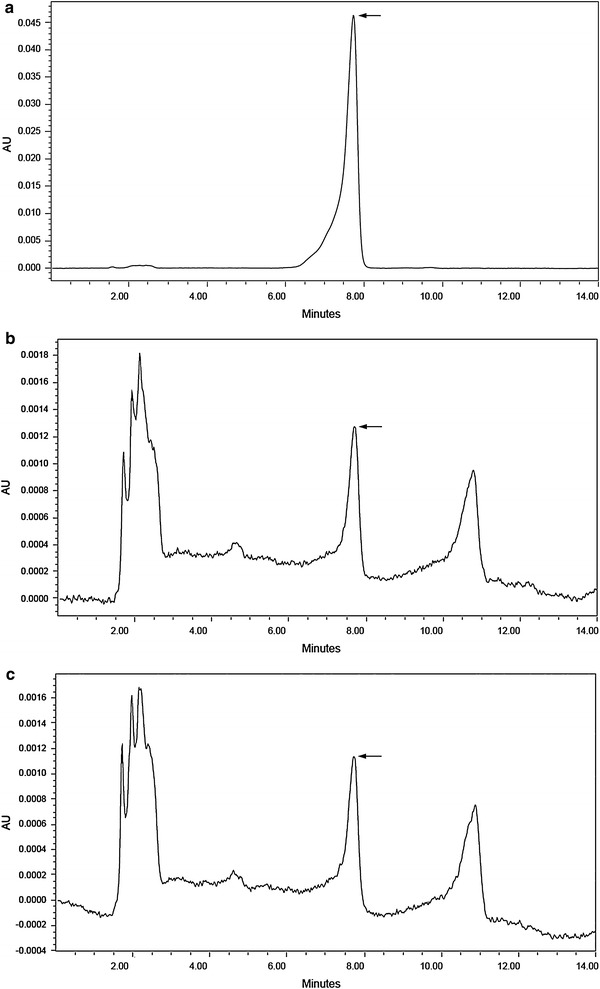
HPLC chromatograms showing AFB1 peaks **a** peak for AFB1 standard, **b** AFB1 peak after degradation by *P. ostreatus* MTCC 142, **c** AFB1 peak after degradation by *P. ostreatus* GHBBF10. AFB1 peaks have been indicated by *arrows*

**Fig. 4:**
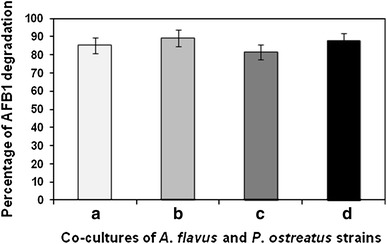
Extent of AFB1 degradation during co-cultivation of fungal strains on rice straw. *a* Straw containing *A. flavus* MTCC 2798 + *P. ostreatus* MTCC 142, *b* straw containing *A. flavus* MTCC 2798 + *P. ostreatus* GHBBF10, *c* straw containing *A. flavus* GHBF09 + *P. ostreatus* MTCC 142, *d* straw containing *A. flavus* GHBF09 + *P. ostreatus* GHBBF10. Data represent mean ± SD (*n* = 3); *P* < 0.05

HPLC chromatogram revealed the peak for AFB1 standard at retention time of 7.722 min (Fig. 3a). The chromatograms for residual AFB1 after degradation by *P. ostreatus* MTCC 142 and *P. ostreatus* GHBBF10 have been illustrated in Fig. 3b and c, respectively. From the chromatograms it became evident that AFB1 was still present in the samples after co-cultivation, but at reduced concentrations. The presence of several peaks other than that for AFB1 may explain the possible formation of AFB1 adducts and various intermediate compounds during AFB1 degradation (Das et al. 2014). The highest degradation of AFB1 (89.41 %) was recorded in the tube containing co-cultures of *A. flavus* MTCC 2798 and *P. ostreatus* GHBBF10, with residual AFB1 content of 2.95 ± 0.03 µg/g (Fig. 4). The lowest degradation (81.73 %) was noted in the tube containing co-cultures of *A. flavus* GHBF09 and *P. ostreatus* MTCC 142, with residual AFB1 content of 3.88 ± 0.01 µg/g. Comparatively, higher percentages of AFB1 degradation were recorded in substrate inoculated with the natural isolate *P. ostreatus* GHBBF10. This could be attributed to its actively functional degradative enzymes’ synthetic machinery owing to its natural isolation from decomposing tree trunk containing lignin. In a previous study on AFB1 degradation in contaminated rice straw by *P. ostreatus* strains, we reported that the natural isolate *P. ostreatus* GHBBF10 secreted higher titres of laccase (1.95 U/gds) and MnP (2.84 U/gds), compared to *P. ostreatus* MTCC 142 (Das et al. 2014). Degradation of recalcitrant compounds by *P. ostreatus* is mediated by secretion of degradative enzymes like laccase and MnP (Bhattacharya et al. 2014). Furthermore, analysis of AFB1 degradation by HPLC and liquid chromatography mass spectrometry revealed reduction in initial concentration of AFB1 and formation of decarbonylated and *O*-dealkylated degradation compounds, respectively (Das et al. 2014).

Due to profuse ramification of the basidiomycete mycelia, the straw containing *A. flavus* MTCC 2798 was effectively colonized and the synthesized AFB1, though present at highest concentration (27.95 ± 0.23 µg/g), was degraded to a greater extent. This observation may be further explained by the presence of yeast extract as nitrogen source in the supplemented mineral salt solution which probably enhanced the laccase production. Gomes et al. (2009) had reported that supplementation of nitrogen in rice straw resulted in 3–4 times increased production of laccase in lignocellulolytic fungi.

In a previous study, co-cultivation of *A. flavus* and *P. ostreatus* was carried out on various substrates. On wheat straw, corn cobs and millet, *A. flavus* produced aflatoxin after 3 weeks of cultivation. A subsequent cultivation of *P. ostreatus* on *A. flavus*-contaminated straw led to detoxification of the straw and corn cobs. It was found that *P. ostreatus* could liquidate colonies of *A. flavus*. However, cultivation of *P. ostreatus* in the presence of 40–100 µg of AFB1/g of substrate did not result in complete detoxification of the material even after 34 days of co-cultivation, but AFB1 concentration decreased to about one-fourth of the added amount (Ginterová et al. 1980).

The present findings are significant as biodegradation of AFB1 with microorganisms or their enzymes may be considered as the best strategy for detoxification of contaminated feedstuffs. This approach is considered as environment friendly in contrast to physico-chemical techniques of detoxification (Upadhaya et al. 2010).

## Conclusions

From this co-cultivation study, it might be deduced that both standard and naturally isolated strains of *P. ostreatus* could effectively degrade moderate concentrations of AFB1 in *A. flavus*-contaminated moldy rice straw. Further studies would be required to enhance the AFB1-degradation potential of the basidiomycete strains through process optimization. 
